# ﻿Two new rarely collected species of Annonaceae from the Peruvian Amazon

**DOI:** 10.3897/phytokeys.262.158372

**Published:** 2025-08-26

**Authors:** Lars W. Chatrou, Sofía Lara-Guerrero, Lennert Gees, Luiz H. M. Fonseca

**Affiliations:** 1 Systematic and Evolutionary Botany lab, Ghent University, K.L. Ledeganckstraat 35, 9000 Ghent, Belgium Ghent University Ghent Belgium; 2 Naturalis Biodiversity Center, Darwinweg 2, 2333 CR Leiden, Netherlands Naturalis Biodiversity Center Leiden Netherlands; 3 Master Program Plant Sciences, IZMB - Institute of Cellular and Molecular Botany, University of Bonn, Kirschallee 1, D-53115 Bonn, Germany University of Bonn Bonn Germany

**Keywords:** *
Klarobelia
*, *
Malmea
*, Neotropics, new species, taxonomy

## Abstract

While preparing a taxonomic revision of the Neotropical genus *Klarobelia* Chatrou (Annonaceae), two species new to science – *Klarobelia icoja* S.Lara & Chatrou, **sp. nov.**, and *Malmea abscondita* Chatrou & Gees, **sp. nov.** – were discovered. Both species are known from only two collections, made in Amazonian Peru.

We clarify their generic placement and taxonomic identity based on a comparison of morphological characters with previously described species and on molecular phylogenetic analysis of four plastid markers.

The conservation status of both species is assessed following IUCN criteria, and line drawings and distribution map are provided.

## ﻿Introduction

For the past three decades, considerable efforts have been made to describe the species diversity of Neotropical Annonaceae, resulting in many monographs, revisions, and ad hoc species descriptions (e.g., Murray 1993; Johnson and Murray 1995; Chatrou and He 1999; Maas et al. 2003, 2007, 2015; Erkens et al. 2017; Pombo et al. 2017; Pirie et al. 2018a; Lopes and Mello-Silva 2019; Bazante et al. 2024). Despite these recent comprehensive taxonomic studies and a global decline in efforts to collect tree species in the Neotropics (ter Steege et al. 2016; Sosef et al. 2017; Luján et al. 2024), new species continue to be discovered.

*Klarobelia* is a small Neotropical genus of Annonaceae that was described following the breakup of the polyphyletic genus *Malmea* (Chatrou 1998). Both genera still contain undescribed species due to the incompleteness of collected material. After the taxonomic revision in 1998, which recognized ten species, three additional species of *Klarobelia* were described – either in the framework of large floristic projects (Chatrou and Pirie 2003; Jørgensen et al. 2014) or to support local conservation efforts (Chatrou and Pirie 2005). As most species of *Klarobelia* are restricted to small areas, new collecting efforts in previously unexplored regions may reveal hitherto unknown species. The discovery of *Klarobelia rocioae* (Maas et al. 2019) – known only from a small area in the Peruvian department of Pasco and first collected in 2003 during a community-based forest monitoring program (Vásquez M. et al. 2005; Kowler et al. 2020) – is a good case in point. Species of *Klarobelia* occur in western lowland Amazonia, Pacific South America, and Central America. The genus is characterized by a combination of the following features: a sunken midvein on the upper side of the leaves, axillary inflorescences, androdioecy, closed flower development, apocarpous fruits with single-seeded monocarps, a raphe that is a sunken, sinuous, or spiral groove on the seed surface, soft seeds with the consistency of cardboard, a striate seed surface, and lamellate ruminations. Species delimitation in *Klarobelia* is mainly based on leaf dimensions; pedicel length in flowering and fruiting stages; dimensions and indument of sepals and petals; dimensions and indument of the fruiting receptacle, monocarps, and stipes; and the shape of the raphe.

Whereas the distribution of *Klarobelia* is essentially centered in the lowland forests surrounding the Andes, *Malmea* has a broader range extending into the Guianas, Central Amazonia, and Brazil’s Atlantic Forest. Like *Klarobelia*, however, all *Malmea* species have narrow, non-overlapping distribution ranges. After the division of *Malmea* into four genera (Chatrou 1998), the six remaining species were collectively represented by approximately 70 specimens, with three species known from only one or two collections. No additional species have been described since that revision. *Malmea* is characterized by the following features: a sunken midvein on the upper side of the leaves, terminal inflorescences, bisexual flowers, open flower development, apocarpous fruits with single-seeded monocarps, a slightly raised raphe, hard seeds with the consistency of glass, a pitted seed surface, and spiniform ruminations. Species delimitation in *Malmea* is mainly based on leaf dimensions; pedicel length in flowering and fruiting stages; dimensions and indument of the fruiting receptacle, stipes, and monocarps; and the shape of the raphe.

Due to their highly localized distributions, many species in these genera have been rarely collected. Several species of *Klarobelia* and *Malmea* are known from no more than four collections. Such infrequently encountered species with small ranges pose significant challenges to plant diversity research in the Neotropics. They are underrepresented in herbarium collections and inventory plot data, making them difficult for taxonomists to describe and for ecologists to include in diversity assessments (Pitman et al. 1999; ter Steege et al. 2013). In this paper, we describe two species of Annonaceae from the Peruvian Amazon that illustrate the challenges of studying this plant group. Both are known from only two specimens, and flowers are unknown for both species. Three of the four collections have been known for over 30 years; the fourth, previously misidentified and filed under *Klarobelia*, was discovered while preparing an update of the genus revision (Chatrou 1998). We feel compelled to describe these species, as undescribed taxa remain a barrier to unlocking baseline biodiversity data. Costello et al. (2013) list several strategies to increase taxonomic productivity, from coordinated field sampling in underrepresented habitats to enhanced interoperability among publications, databases, and scholarly platforms. Although their list is extensive, we suggest that describing new species despite incomplete morphological knowledge may also accelerate taxonomic progress.

Despite the paucity of specimens and the absence of flowers, the collections clearly represent distinct, new species of *Klarobelia* and *Malmea*. Like most genera in tribe Malmeeae, these genera are defined by unique combinations of morphological characters (as outlined above), each of which individually displays high levels of homoplasy within the family. As these genera may be difficult for non-specialists to recognize, we supplement the species descriptions with phylogenetic analyses to demonstrate their placement within the monophyletic genera *Klarobelia* and *Malmea*, respectively.

## ﻿Methods

Species descriptions are based on herbarium specimens from the Missouri Botanical Garden (MO), Field Museum of Natural History (F), and the herbarium of Utrecht University (U), now incorporated into the collection of Naturalis Biodiversity Center, Leiden, the Netherlands. The descriptions include only measurements taken from dried specimens. Line drawings were produced by SLG by digitizing specimens using Procreate version 5.3.7 on an iPad (9^th^ generation) and then elaborating with ink on paper.

The distribution map was generated using QGIS 3.34.11, based on specimen data from F, U, and MO. Departmental borders in Perú were sourced from the Instituto Geográfico Nacional, as published on Plataforma Nacional de Datos Abiertos (https://www.datosabiertos.gob.pe/dataset/limites-departamentales). Coordinates for the paratype of the new *Malmea* species were georeferenced using the Georeferencing Calculator (Wieczorek and Wieczorek 2021), based on Zermoglio et al. (2020). Type specimens are referenced by their herbarium barcode numbers. The GeoCAT geospatial conservation assessment tool (Bachman et al. 2011) was used for the preliminary conservation assessment of the described species. The area of occupancy (AOO) was calculated using a cell size of 2 km, in accordance with Criterion B of the IUCN Red List (IUCN Standards and Petitions Committee 2024). As both species are known from only two specimens, a polygon encompassing these data points could not be drawn, and therefore the extent of occurrence (EOO) would be calculated as 0. Following the recommendation of the IUCN Standards and Petitions Committee (2024), EOO was set equal to AOO to maintain consistency with the definition of AOO as an area within EOO.

### ﻿Molecular phylogenetic analyses

For the phylogenetic analysis, a supermatrix was compiled comprising 40 species of tribe Malmeeae (Chatrou et al. 2018; Nge et al. 2024; Helmstetter et al. 2025) and three outgroup species from subfamilies Ambavioideae and Annonoideae. Sequence data for plastid markers (*rbcL*, *trnLF*, *matK*, *psbA-trnH*) were included, most of which have been published previously (Pirie et al. 2005, 2006; Chatrou et al. 2012; Thomas et al. 2015). The new *Malmea* species described in this paper was included in earlier phylogenetic analyses as ‘*Malmea* sp. 0197’ (Pirie et al. 2006, 2007; Chatrou et al. 2012; Pirie et al. 2018a, 2018b). For the new *Klarobelia* species, we sequenced *rbcL* (GenBank accession PV861990), *trnLF* (PV861992), and *matK* (PV861991), following methods described by Pirie et al. (2006), using DNA extracted from Mathias & Taylor 5036.

The four alignments – *rbcL* (1,376 positions), *trnLF* (937 positions), *matK* (831 positions), and *psbA-trnH* (531 positions) – were analyzed separately using Bayesian phylogenetic inference performed with MrBayes 3.2.7 (Ronquist et al. 2012), available on the CIPRES portal in San Diego, CA, USA (http://www.phylo.org/portal2; Miller et al. 2010). For all analyses, DNA substitution models and phylogenetic parameters (e.g., topology, branch lengths) were estimated simultaneously using a reversible jump Markov chain Monte Carlo sampler (model-jumping; Huelsenbeck et al. 2004), allowing among-site rate heterogeneity (Γ). The MCMC chain was initially run for 25 million generations, with four simultaneous runs and four chains per run, using default settings for the acceptance rates and the temperature of the heated chains. Sampling occurred every 1,000 generations. Convergence diagnostics were assessed using the *sump* command in MrBayes and by examining ESS values in Tracer.

After confirming the absence of incongruence among the phylogenetic trees derived from the separate marker analyses, 55 indel characters derived from the *trnLF* alignment were added and scored using the simple indel coding method (Simmons and Ochoterena 2000). A concatenated analysis of the five data partitions was then performed using the same settings as above, with the addition of the binary model (lset coding = variable; Lewis 2001) for the indel characters. After convergence was reached, maximum clade credibility trees were generated using LogCombiner and TreeAnnotator in BEAST (Drummond and Rambaut 2007).

Maximum likelihood bootstrap analyses, implementing the rapid heuristic bootstrap method described by Stamatakis et al. (2008), were conducted using RAxML v.8.2.12 (Stamatakis 2014).

## ﻿Results and discussion

### ﻿Molecular phylogenetic analyses

**Figure 1.**
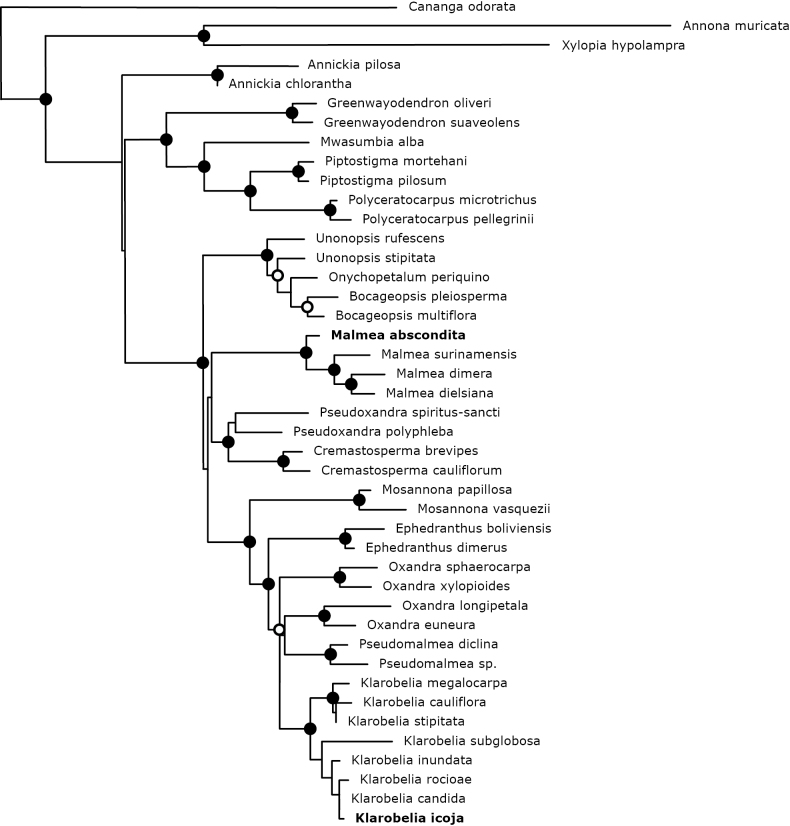
Phylogenetic position of *Malmea abscondita* and *Klarobelia icoja.* Best tree resulting from an RAxML analysis (best LH = -11766.962161) of a supermatrix of four plastid markers. Closed circles indicate Bayesian posterior probabilities ≥ 0.95 and RAxML bootstrap percentage ≥ 85%. Open circles indicate Bayesian posterior probabilities (≥ 0.95) only.

Bayesian phylogenetic inference of the four-marker dataset resulted in convergence of the two runs on equal posterior probability distributions, with sample sizes sufficient as indicated by the ESS values. The best tree from the RAxML analysis, that is, the tree with the highest likelihood value (best LH = –11766.962161), is shown in Fig. 1. Due to reduced species sampling, some support values were lower than those in previously published analyses based on the same data (e.g., Pirie et al. 2006). The principal result is the re-establishment of the well-supported monophyly of *Klarobelia* and *Malmea*, which justifies the inclusion of the new species in these genera.

### ﻿Morphological characters

Despite the absence of flowers in both species, the presence of leaves, fruits, and seeds is sufficient to assign the two new species to *Klarobelia* and *Malmea*, respectively, and to distinguish them from their congeners. For *Klarobelia*, diagnostic features include general leaf morphology, the overall ‘gestalt’ of the fruit, and in particular the soft seeds with a sinuous groove-like raphe and lamellate ruminations. For *Malmea*, the key identifying features are general leaf morphology; terminal inflorescences, which are rare in Neotropical Annonaceae with apocarpous fruits; and seeds with a pitted surface and spiniform ruminations. Details on the characters that distinguish these species from their most similar congeners are provided in the diagnoses below.

### ﻿Taxonomic treatment

#### ﻿ Klarobelia icoja

Taxon classificationPlantaeMagnolialesAnnonaceae

S.Lara & Chatrou sp. nov.

1665E8DF-2F5F-52B9-894B-B65FE4ACBBD3

urn:lsid:ipni.org:names:77368248-1

Fig. 2

##### Type.

Peru • [Dept. Ucayali: Province Padre Abad], vicinity of Aguaytía, [09°03'S, 075°30'W], 22 Jun 1961, *M.E. Mathias & D. Taylor 5390* (holotype: MO! [barcode MO-5509445]; isotype: F! [barcode V0205843F]).

##### Diagnosis.

*Klarobelia icoja* resembles *K. lucida* in the size of the monocarps and the thin fruit wall and *K. pumila* in the leaves, but it can be distinguished from *K. lucida* by the leaves with a length up to 19 cm (vs. 13 cm), narrower leaves with a length-width ratio of 3.3–3.5 (vs. 1.8–2.8), acute base (vs. obtuse to acute), and shorter pedicels of 12–17 mm long (vs. 17–68 mm long). *K. icoja* can be distinguished from *K. pumila* by the monocarps, which are longer (19–22 mm vs. 9–15 mm) and wider (10–11 mm vs. 6–8 mm), and by the longer stipes (28–37 mm vs. 12–20 mm).

##### Description.

Tree up to 4 m tall. Young twigs and developing leaves sparsely covered with yellowish-brown, appressed hairs. Petiole 4–8 mm long, 1.0–1.5 mm wide, verrucose, glabrous. Lamina 11.3–19.0 cm long, 3.5–5.7 cm wide, length-width ratio 3.3–3.5, chartaceous, slightly bullate, elliptic, base acute, apex gradually acuminate, light olive green above and dark olive green below, glabrous above and below, primary vein impressed (to flat) above, 6–9 secondary veins per side, distance between secondary veins 11–22 mm, angles with primary vein 60–75°, loop-forming at right to obtuse angles, distance between loops and leaf margin 4–7 mm. Inflorescences ramiflorous, on the leafless part of the twig, single-flowered. Short shoot 2–3 mm long, 2.0–2.5 mm in diam. when fruiting. Bracts not observed. Pedicels 12–17 mm long, 1.5–2.0 mm in diam. (in fruit). Fruit of ca. 10 monocarps (scars suggest up to ca. 25 monocarps), orange-red in vivo, reddish brown to black in sicco, ellipsoid, 19–22 mm long, 10–11 mm in diam., glabrous, verrucose, raphe visible through fruit wall, stipes 28–37 mm long, 1 mm in diam., fruiting receptacle subglobose, 7–9 mm in diam., 4–6 mm high. Seed ellipsoid, 21 mm long, 10 mm in diam., brownish-orange, raphe a sinuous groove, ruminations in four lamellate parts.

**Figure 2.**
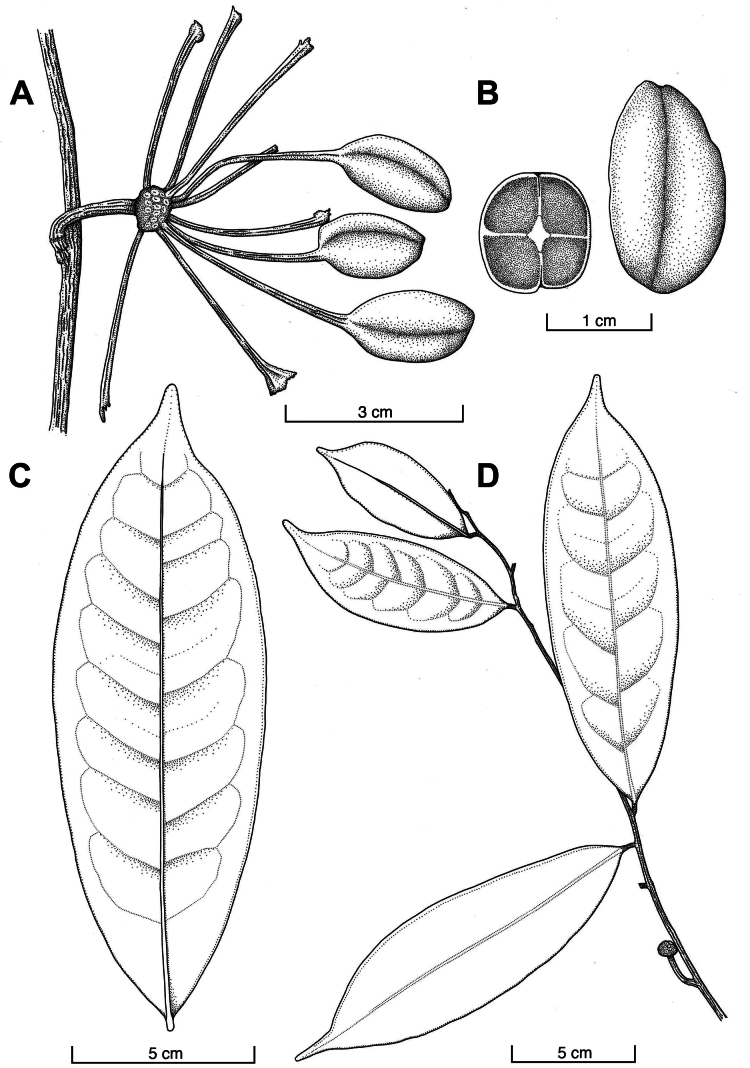
*Klarobelia icoja* S.Lara & Chatrou. **A.** Habit with fruit; **B.** Monocarp (right) and cross section of seed (left), showing the ruminations in four lamellate parts; **C**; Leaf; **D.** Habit with fruiting receptacle. (**A, C, D.***Mathias & Taylor 5390*; **B.***Mathias & Taylor 5036*).

##### Local names.

Peru: Jicoja or Icoja, based on the holotype label.

##### Distribution and ecology.

**Figure 3.**
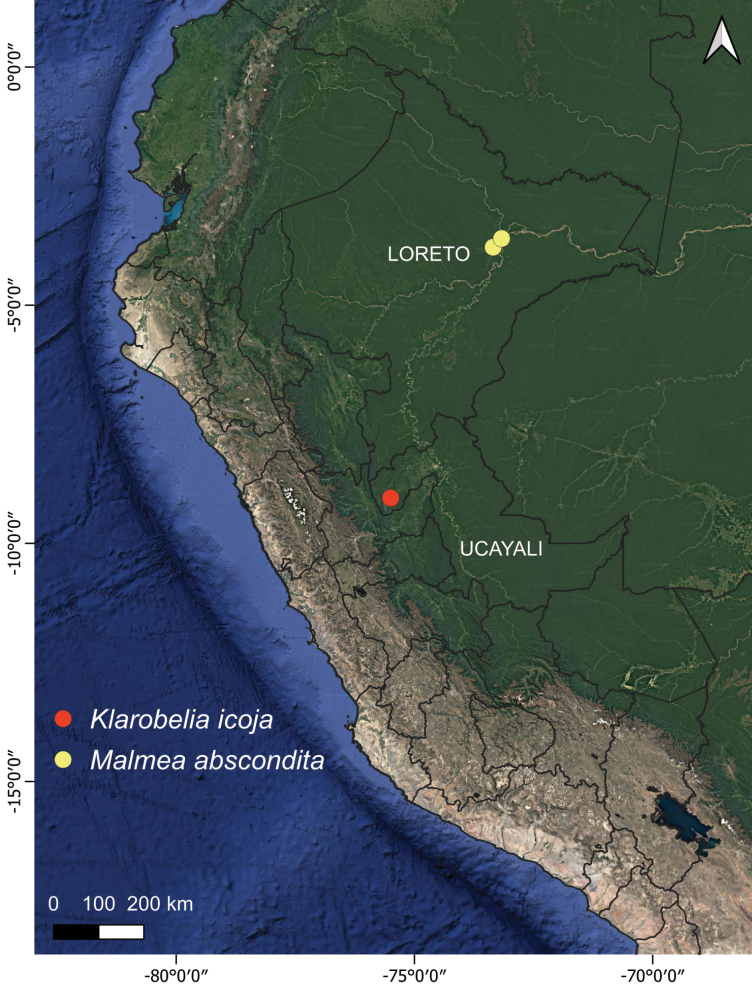
Distribution map of *Klarobelia icoja* S.Lara & Chatrou and *Malmea abscondita* Chatrou & Gees.

The species is known from the northern part of Ucayali, in the vicinity of Aguaytía, Perú (Fig. 3; only the locality of the type specimen is presented here, given the absence of latitude-longitude data of the second specimen). It grows in forests; information about its elevation range is unknown. The label of Mathias & Taylor 5036 mentions that the fruits are orange-red. Most monocarps in both collections have been removed while the stipe has remained on the fruit. In Annonaceae, monocarps that detach from fruit do so with the stipe attached to the monocarp, leaving scars of the stipe on the fruiting receptacle. The detachment of the monocarp only in this species is indicative of zoochory.

##### Preliminary conservation status.

The label of the type specimen does not mention latitude and longitude. We took these from an earlier collection by the same collector on the same day (Mathias & Taylor 5368, *Miconia alternidomatia* Michelang.). To date, *Klarobelia icoja* is known only from two collections made over 50 years ago in two consecutive years in the vicinity of Aguaytía, with an AOO and EOO of 4 km^2^. It falls under the IUCN category of data deficient (DD). A more detailed analysis of the population must be done to correctly assess its conservation status. Given the general geographical pattern of *Klarobelia*, characterized by small allopatric distributions, this is likely to be accomplished by more thorough inventories of the known area of occurrence.

##### Etymology.

The specific epithet refers to the indigenous name of the species. “Icoja,” “hicoja,” or “jicoja,” however, is not unique to this species and refers to Annonaceae in general, as it has been reported for Peruvian species in the genera *Anaxagorea* (Maas and Westra 1984), *Bocageopsis* (Maas et al. 2007), *Cremastosperma* (Pirie et al. 2018a), *Fusaea* (Chatrou and He 1999), *Guatteria* (Maas et al. 2015), and *Unonopsis* (Maas et al. 2007). The name *icoja* (pronunciation in International Phonetic Alphabet: i'koxa) is used by the indigenous Quechua people (Sanz-Biset et al. 2009). This is suggesting the name may stem from Quechua or Runa simi, the indigenous language family that originated in central Peru, even though Sanz-Biset et al. (2009) indicate that indigenous Quechua plant names have been mixed with Spanish to varying degrees.

##### Additional specimen examined.

Peru • [Ucayali: Province Padre Abad], vicinity of Aguaytía, high ground in forest southeast of house, don Diogenes del Aguila, east of Aguaytía, between Pucallpa road and Aguaytía river, 29 Jun 1960, M.E. Mathias & D. Taylor 5036 (MO!).

#### ﻿ Malmea abscondita

Taxon classificationPlantaeMagnolialesAnnonaceae

Chatrou & Gees sp. nov.

229BF9BA-3352-5148-A9AA-342444504603

urn:lsid:ipni.org:names:77368249-1

Fig. 4

##### Type.

Peru • Dept. Loreto: Río Nanay, San Pablo de Cuyana, Estación Biológica Miguel Alejandro, 03°47'S, 73°21'W, 9 Nov 1994, *L.W. Chatrou, P.J.M. Maas, H. Rainer & F. Ayala 8* (holotype: USM! [specimen number USM-135298]; isotype: U! [barcode U-0089935, U-0089936]).

##### Diagnosis.

*Malmea abscondita* is distinct from congeneric species by a combination of the following characters: narrowly elliptic leaves, few secondary veins, fewer monocarps, and longer stipes of the monocarps. The new species combines characters mostly found in *Malmea guianensis* R.E.Fr., *M. manausensis* Maas & J.M.S. Miralha, and *M. surinamensis* Chatrou. It differs from *Malmea guianensis* by the hairy inflorescences (vs. glabrous), the smaller number of monocarps (6–13 vs. 20), and shorter stipes (22–30 mm vs. 37–49 mm); from *M. manausensis* by the lower number of secondary veins (8–9 vs. 12–14 per side of the leaf), the longer and more slender stipes (22–30 mm long, 1 mm in diam. vs. 18–21 mm long, 2.5 mm in diam.); and from *M. surinamensis* by the lower number of secondary veins (8–9 vs. 10–13 per side of the leaf), the longer pedicels (43–57 mm vs. 40 mm), and the smaller number of monocarps (6–13 vs. 15–20).

##### Description.

Small tree, 2.5–4 m tall, 2 cm in diam. Young twigs and lower side of petiole and basal part of midrib sparsely to rather densely covered with brown, appressed (to erecto-patent) hairs ca. 0.5 mm long (simply hairs hereafter). Petiole 4–6 mm long, 1.5–2 mm in diam., verrucose to rugulose. Lamina 19–30 cm long, 5–8 cm wide, length-width ratio (2.9–)3.3–4.2, chartaceous, narrowly elliptic or narrowly obovate, base cuneate to obtuse, apex gradually acuminate, olive green and shiny above, olive green (slightly darker than upper side) below, glabrous above, (sub-)glabrous below, secondary veins 8–9 per side, irregularly spaced with distance between secondary veins 14–35 mm, angles with primary vein 55–75°, loop-forming at right to obtuse angles, distance between loops and leaf margin 5–10 mm. Flowers unknown. Infructescences on leafy twigs, terminal or leaf-opposed, maximally 1 flower/fruit scar and 1 actual fruit on rhipidium, peduncles and pedicels sparsely to rather densely covered with short, yellowish-brown, appressed to erecto-patent hairs, bract densely covered with similar hairs. Peduncles 10 mm long, 1–2 mm in diam. Pedicels 43–57 mm long, 1 mm in diam. basally to 2.5 mm apically, sympodial rachis 3 mm long. Articulation 1 mm above axil of lower bract. Bracts 2.5 mm long, 2 mm wide, apex obtuse to rounded, semi-amplectent, lower bract near base of pedicel, upper bract halfway the pedicel. Fruit of 6–13 monocarps, blackish brown, ellipsoid, 13–16 mm long, 8–11 mm in diam., sparsely hairy except for densely hairy apex, verrucose; stipes blackish brown, 22–30 mm long, 1 mm in diam., sparsely to rather densely hairy; fruiting receptacle (sub)globose, 6–8 in diam., 6–9 mm high, (rather) densely hairy. Seeds ellipsoid, 12–15 mm long, 7–10 mm in diam., brown, surface pitted, raphe a slightly elevated rib, ruminations spiniform.

**Figure 4.**
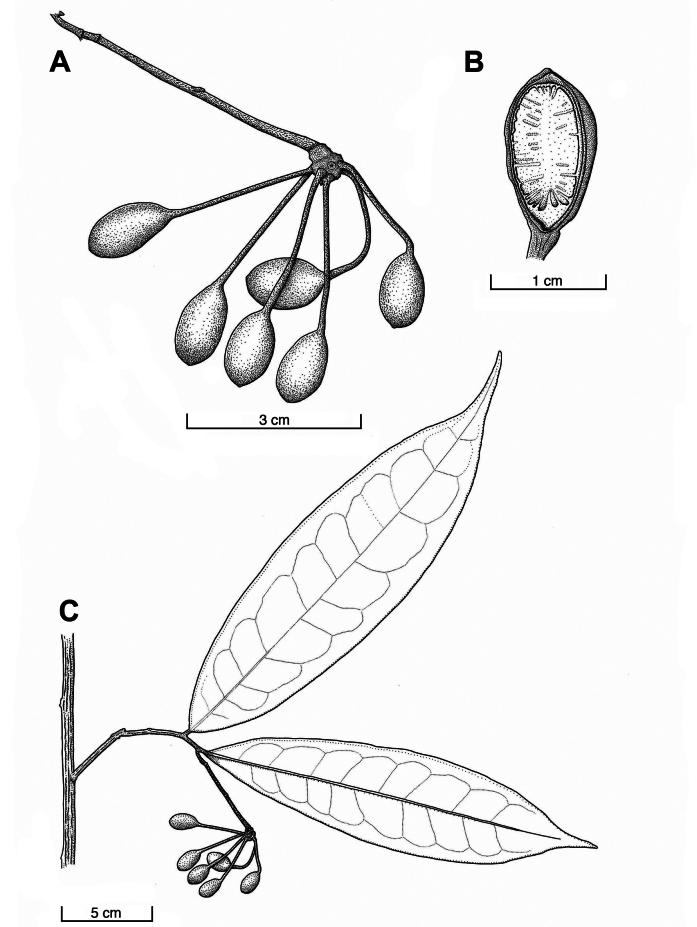
*Malmea abscondita* Chatrou & Gees. **A.** Fruit; **B.** Longitudinal section of monocarp, showing spiniform ruminations; **C.** Habit with fruit. (**A–C.***Chatrou et al. 8*).

##### Distribution and habitat.

The species is known from two localities near Río Nanay, west to northwest of Iquitos, in the Peruvian department of Loreto (Fig. 3). Both the occurrence in upland rainforest and periodically inundated rainforest have been reported.

##### Preliminary conservation status.

To date, *Malmea abscondita* is known only from two collections made in the vicinity of Río Nanay, with an AOO and EOO of 8 km^2^. The collection label of Rimachi 479 does not include latitude and longitude, and we estimated the collection locality to be just north of the mouth of Río Nanay, near the *carretera Nanay – Mazan*. The species falls under the IUCN category of data deficient (DD). A more detailed analysis of the population must be done to correctly assess its conservation status. Given the general geographical pattern of *Malmea*, characterized by small allopatric distributions, this is likely to be accomplished by more thorough inventories of the known area of occurrence.

##### Etymology.

The specific epithet refers to the concealed, largely unnoticed existence of this species in the Amazonian forests of northern Peru, having been collected twice only in the past 52 years.

##### Additional specimen examined.

Peru • [Loreto], Maynas, Trocha de la Astoria hasta Mazan, near mouth of Río Nanay, 2 Aug 1973, M. Rimachi Y. 479 (MO!).

## Supplementary Material

XML Treatment for Klarobelia icoja

XML Treatment for Malmea abscondita

## ﻿Additional information

### Use of AI

No use of AI was reported.

### Author ORCIDs

Lars W. Chatrou https://orcid.org/0000-0003-0131-0302

Sofía Lara Guerrero https://orcid.org/0000-0001-6845-6408

Luiz H. M. Fonseca https://orcid.org/0000-0002-8140-3244

### Data availability

All of the data that support the findings of this study are available in the main text.
